# Availability, prices, and affordability of essential medicines for cardiovascular diseases and diabetes: a multi-regional survey using the WHO/HAI methodology in Pakistan

**DOI:** 10.1186/s12913-026-14792-9

**Published:** 2026-06-06

**Authors:** Muhammad Uzair Ul Haq, Saima Mushtaq, Smavia Jamshed, Oshna Quddoos, Aimen Rija Tareen, Shehroz Ahmad Khan, Qadeer Ahmad, Amjad Khan, Yu Fang

**Affiliations:** 1 Department of Pharmacy, Quaid-i-Azam University, Islamabad, Pakistan; 2 Department of Pharmacy, The First Affiliated Hospital of Xi’an Jiaotong University, Xi’an, China; 3 Department of Pharmacy Administration and Clinical Pharmacy, School of Pharmacy, Health Science Center, Xi’an Jiaotong University, Xi’an, China

**Keywords:** Essential medicines, WHO/Health Action International (WHO/HAI) methodology, Drug availability, Drug affordability, Median price ratio, Cardiovascular diseases, Diabetes mellitus, Pakistan

## Abstract

**Background:**

Ensuring equitable access to essential medicines is fundamental to universal health coverage, yet disparities in availability, pricing, and affordability persist in many low- and middle-income countries.

**Objectives:**

This study assessed the availability, prices, and affordability of essential cardiovascular and antidiabetic medicines across public and private healthcare sectors in Pakistan using the WHO/Health Action International (WHO/HAI) methodology.

**Methods:**

A cross-sectional survey of 32 essential medicines was conducted across selected study areas (Rawalpindi, Muzaffargarh, Vehari, Gujranwala, Islamabad, and Azad Jammu & Kashmir). Medicine availability was measured as the percentage of facilities stocking medicines on the survey day. Prices were analyzed using median price ratios (MPRs) against international reference prices, and affordability was calculated as the number of days’ wages required by the lowest-paid unskilled government worker to purchase a standard 30-day treatment. Sectoral and regional differences were evaluated using chi-square tests.

**Results:**

The availability of the 32 cardiovascular and antidiabetic medications was generally poor, especially in government facilities; originator brands (OBs) and lowest-priced generics (LPGs) were consistently more readily available in private pharmacies (χ²=180.6, *p* < 0.001). There were notable geographical differences, with rural areas exhibiting very poor access (χ²=202.8, *p* < 0.001) and Gujranwala exhibiting the greatest availability. While certain OBs, particularly insulin analogs, were noticeably more expensive than LPGs, prices and affordability favored LPGs across all medications. Public and private sectors continued to have insufficient supplies of insulin and emergency medications, falling short of WHO objectives.

**Conclusion:**

This study reveals persistent inequities in the availability and affordability of essential medicines across the selected districts in Pakistan. While these findings specifically reflect the studied regions, they provide actionable evidence to strengthen Pakistan’s public-sector supply systems, promote generic medicines, and inform national access policies.

## Introduction

The global burden of non-communicable diseases (NCDs), particularly cardiovascular diseases (CVDs) and diabetes, has risen substantially, accounting for over 70% of deaths worldwide, with more than 85% occurring in low- and middle-income countries (LMICs) [1]. Between 1990 and 2019, CVD prevalence increased from 3304.2 to 4944.1 cases per 100,000 population, while mortality rose from 139.8 to 182.1 per 100,000 [2]. Essential medicines play a critical role in addressing this burden by preventing complications, reducing mortality, and improving quality of life. Ensuring access to safe, effective, high-quality, and affordable medicines is therefore a cornerstone of Universal Health Coverage (UHC) and is emphasized under Sustainable Development Goal (SDG) 3.8 [3].

Access to essential medicines is widely recognized as a fundamental component of the right to health, particularly in LMICs where health system resources are constrained [4]. The World Health Organization (WHO) Model List of Essential Medicines (EML) provides a globally accepted framework for prioritizing medicines based on disease prevalence, safety, efficacy, and cost-effectiveness [5]. WHO further emphasizes that these medicines should be consistently available, in appropriate dosage forms, and at affordable prices across all levels of care [6]. The WHO and Health Action International (HAI) developed a globally validated method in 2003 to assess the costs, accessibility, and affordability of medications across all healthcare sectors due to the need for consistent and comparable evidence to inform pharmaceutical policy [6]. Despite these commitments, more than one-third of the global population lacks reliable access to essential medicines, largely due to high costs, inefficient supply chains, weak procurement systems, and limited regulatory oversight [6–9].

In Pakistan, the burden of NCDs is rapidly increasing, creating substantial demand for long-term pharmacotherapy. Cardiovascular-related deaths nearly doubled from 172,526 in 1990 to 341,108 in 2019, with disability-adjusted life years (DALYs) rising from 4.5 million to 9.8 million [10]. Similarly, the number of individuals with diabetes increased dramatically from 5.2 million in 2000 to approximately 33 million in 2021 [11]. This epidemiological transition highlights the urgent need to ensure equitable access to essential cardiovascular and antidiabetic medicines to reduce complications, hospitalizations, and premature mortality. Pakistan’s pharmaceutical policy framework is guided by the National Essential Medicines List (NEML), adapted from the WHO EML to reflect national health priorities [12]. While inclusion in the NEML is intended to facilitate efficient procurement and rational prescribing, evidence suggests that it does not guarantee medicine availability or affordability, particularly in under-resourced public health facilities affected by stock-outs and supply chain inefficiencies [13]. Data shows that national price deregulation failed to improve antidiabetic drug access, with mean availability at 72% (well below the 80% WHO target) and costs remaining unaffordable for LPGW earners [14]. Furthermore, although essential medicines are cost-effective in the long term, their affordability remains a major concern, as improved adherence has been associated with reductions of 36% in emergency visits and 44% in hospitalizations [15, 16].

Significant disparities persist in Pakistan regarding the availability and affordability of essential medicines. The availability of cardiovascular medications is still well behind the WHO’s 80% target in both sectors, according to a 2021 WHO/HAI survey. While originator brands were more accessible in the private sector (54.6% vs. 34.9%), lowest-price generics were somewhat more accessible in the public sector (30.4% vs. 25.5%); overall, availability for both categories remained below ideal [17]. Treatment expenses often surpass patients’ daily earning potential. For instance, a month’s supply of cardiovascular medications may cost up to 21.5 days’ earnings (USD 81.56) for originator-brand lovastatin and about 4.1 days’ salaries (USD 1.54) for the cheapest generic captopril [1]. Essential anti-diabetic medications have similar problems. According to a survey in Pakistan in 2019, no insulin product met the WHO’s 80% availability target, according to survey data; public-sector availability stayed below 50%, while private-sector availability varied from 62.5% to 90%, with several products still falling short of the suggested cutoff. As a result, patients frequently depend on pricey imported insulin products, which has an impact on equity and affordability [14, 18]. These issues are compounded by high out-of-pocket (OOP) expenditures, which account for 60–70% of total health spending in Pakistan [19]. The Sehat Sahulat Program (SSP), launched in 2015, aims to provide financial risk protection by offering inpatient coverage of up to PKR 1 million (≈ USD 3,600) through a network of over 400 hospitals. The program has expanded substantially, now covering over 10 million families nationwide, with a marked increase in utilization following universal rollout [20, 21]. Although initiatives such as the SSP aim to enhance financial protection and expand healthcare access, their effectiveness depends heavily on the consistent availability and affordability of essential medicines [20–22].

Given these challenges, there is a critical need for robust, context-specific evidence on medicine prices, availability, and affordability. The present study applies the standardized WHO/Health Action International (HAI) methodology to evaluate these parameters for selected cardiovascular and antidiabetic medicines across public and private sectors in Pakistan. By identifying gaps and inefficiencies, this research aims to inform pharmaceutical policy, support UHC implementation, and improve equitable access to essential medicines in Pakistan.

## Methodology

### Study design

A cross-sectional, descriptive methodology was used in this study to assess the availability, pricing, and affordability of critical cardiovascular and antidiabetic medications in Pakistan. The study methodology was based on the World Health Organization/Health Action International (WHO/HAI) methodology which provides a standardized and internationally comparable framework for the assessment of availability, prices and affordability of medicines.

### Study setting and health system context

Pakistan’s public healthcare system operates through a decentralized, three-tiered structure aimed at achieving universal health coverage. Primary care facilities, including Basic Health Units (BHUs) and Rural Health Centers (RHCs), serve as the first point of contact and provide basic outpatient and emergency services. Secondary care comprises Tehsil and District Headquarter hospitals (THQs and DHQs), which offer specialized diagnostic and treatment services, while tertiary care is delivered through large teaching and specialized hospitals located in major urban centers.

The study was conducted across six regions: Islamabad Capital Territory, four districts of Punjab Province (Rawalpindi, Gujranwala, Muzaffargarh, and Vehari), and Azad Jammu and Kashmir. These locations were purposively selected to capture geographic and socioeconomic diversity, representing northern (Islamabad, Rawalpindi, AJK), central (Gujranwala), and southern Punjab (Muzaffargarh, Vehari). The inclusion of urban, peri-urban, and rural settings, along with variation in healthcare infrastructure and service delivery, allows for a comprehensive assessment of medicine availability and affordability across heterogeneous regions of Pakistan.

In Pakistan, noncommunicable diseases like cardiovascular diseases and diabetes are managed through primary care for initial diagnosis and basic treatment, while secondary and tertiary hospitals handle complications. Due to regular medicine shortages in public facilities, many patients depend on private pharmacies for ongoing medication.

### Sampling strategy

The sampling strategy adhered to WHO/HAI project criteria, selecting a total of 60 health outlets across six survey regions (10 outlets per region). Each region included 5 public hospitals and 5 private community pharmacies. Public hospitals were selected using a stratified approach based on care level: primary care (Basic Health Units [BHUs] and Rural Health Centers [RHCs], providing first-contact outpatient and emergency services), secondary care (Tehsil Headquarters [THQ] and District Headquarters [DHQ] hospitals, offering specialized diagnostic and treatment services), and tertiary care (large teaching and specialty hospitals in major urban centers). Within each region, one tertiary, two secondary, and two primary care facilities were purposively selected to capture variation in medicine procurement, stock-out patterns, and logistics management. Private pharmacies were sampled within a 10 km radius of the public facilities to enable direct sectoral comparison of medicine prices and availability across urban, peri-urban, and rural catchment areas.

### Data collection and sources

Data on medicine availability and prices were obtained from stock records and direct observation at surveyed health facilities. Pharmacy personnel, including pharmacists, pharmacy assistants, and store managers responsible for medicine dispensing and inventory management, facilitated access to inventory and pricing information during the data collection process; however, they were not research participants, and no personal data or qualitative information were collected from them.

### Medicine selection

A total of 32 essential medications were chosen for the study, with an emphasis on diabetes and cardiovascular diseases, based on the Sehat Sahulat Universal Health Coverage package, the 23rd edition (2023) of the WHO Essential Medicines List (EML), which is updated every two years, and the 23rd edition (2023) of the Pakistan National Essential Medicines List (NEML), which was created in accordance with the WHO framework and updated with similar frequency. Due to the large and rising prevalence of cardiovascular diseases and diabetes mellitus in Pakistan, and their need for long-term pharmacotherapy, these medications were given priority since they are essential for evaluating access, affordability, and the effectiveness of the health system. The list includes high-priority classes such as ACE inhibitors, Statins, Beta-blockers, Biguanides (Metformin), and Insulins, which are critical for preventing complications. The medicines were categorized into three distinct baskets reflecting healthcare levels: a Primary Care basket (14 medicines), a Secondary Care basket (14 medicines), and a Tertiary Care basket (6 medicines). To align with the prescribing capacity and stocking norms of different facility types, a stratified survey approach was employed: the full list of 32 medicines was assessed at the tertiary care hospital, a subset of 26 medicines (combining Primary and Secondary baskets) was surveyed at secondary care hospitals, and only the Primary basket of 14 medicines was assessed at primary care facilities (Basic Health Units and Rural Health Centers) (see Table 1). On the day of the survey, information was gathered regarding the availability and cost of the originator brand (OB) and the lowest-priced generic (LPG) equivalent of each medication.

**Table 1:** Selected essential cardiovascular and diabetes medicines surveyed using the WHO/HAI methodology, including strength, dosage form, therapeutic category, and therapeutic class

Medicine	Strength	Dosage Form	Therapeutic Category	Therapeutic class	*P*	S	T
Aspirin	75 mg	Tab	Cardiovascular	Antiplatelet agent	Yes	Yes	Yes
Atenolol	50 mg	Tab	Cardiovascular	β-blocker	Yes	Yes	Yes
Atorvastatin	10 mg	Tab	Cardiovascular	Statin	Yes	Yes	Yes
Captopril	25 mg	Tab	Cardiovascular	ACE inhibitor	Yes	Yes	Yes
Captopril	50 mg	Tab	Cardiovascular	ACE inhibitor	Yes	Yes	Yes
Propranolol	40 mg	Tab	Cardiovascular	β-blocker	Yes	Yes	Yes
Glyceryl Trinitrate	0.5 mg	Tab	Cardiovascular	Nitrate	Yes	Yes	Yes
Nifedipine	20 mg	Tab	Cardiovascular	Calcium channel blocker	Yes	Yes	Yes
Lisinopril	10 mg	Tab	Cardiovascular	ACE inhibitor	Yes	Yes	Yes
Losartan	50 mg	Tab	Cardiovascular	Angiotensin receptor blocker	Yes	Yes	Yes
Metoprolol	100 mg	Tab	Cardiovascular	β-blocker	Yes	Yes	Yes
Amlodipine	5 mg	Tab	Cardiovascular	Calcium channel blocker	Yes	Yes	Yes
Valsartan	80 mg	Tab	Cardiovascular	Angiotensin receptor blocker	Yes	Yes	Yes
Verapamil Hydrochloride	40 mg	Tab	Cardiovascular	Calcium channel blocker	Yes	Yes	Yes
Dobutamine	250 mg / 20 ml	Vial	Cardiovascular	Inotropic agent	No	Yes	Yes
Adenosine	3 mg / ml	Vial	Cardiovascular	Antiarrhythmic	No	Yes	Yes
Amiodarone	200 mg	Tab	Cardiovascular	Antiarrhythmic	No	Yes	Yes
Bisoprolol	5 mg	Tab	Cardiovascular	β-blocker	No	Yes	Yes
Carvedilol	25 mg	Tab	Cardiovascular	β-blocker	No	Yes	Yes
Furosemide	40 mg	Tab	Cardiovascular	Loop diuretic	No	Yes	Yes
Metformin	500 mg	Tab	Diabetes	Biguanide	No	Yes	Yes
Insulin Degludec	100 IU/mL	Vial	Diabetes	Long-acting Insulin	No	Yes	Yes
Insulin Detemir	100 IU/mL	Vial	Diabetes	Long-acting Insulin	No	Yes	Yes
Insulin Glargine	100 IU/mL	Vial	Diabetes	Long-acting Insulin	No	Yes	Yes
Insulin NPH	100 IU/ ml	Vial	Diabetes	Intermediate-acting Insulin	No	Yes	Yes
Empagliflozin	10 mg	Tab	Diabetes	SGLT-2 inhibitor	No	Yes	Yes
Insulin Regular	100 IU/ ml	Vial	Diabetes	Short-acting insulin	No	No	Yes
Fluvastatin	20 mg	Tab	Cardiovascular	Statin	No	No	Yes
Clopidogrel	75 mg	Tab	Cardiovascular	Antiplatelet agent	No	No	Yes
Enoxaparin	40 mg / 0.4 ml	Vial	Cardiovascular	Anticoagulant	No	No	Yes
Digoxin	250 mcg (0.25 mg)	Tab	Cardiovascular	Cardiac glycoside	No	No	Yes
Streptokinase	1.5 million IU	Vial	Cardiovascular	Thrombolytic agent	No	No	Yes

Abbreviations: P = Primary basket; S = Secondary basket; T = Tertiary basket. “Yes” indicates inclusion of the medicine in the respective basket, while “No” indicates non-inclusion

### Data collection

Trained data collectors conducted the survey from October to mid-November 2025 using a standardized WHO/HAI data collection tool, adapted into a customized medicine form to capture essential cardiovascular and antidiabetic medicines specific to the study regions. Data was gathered by direct observation of medication stocks and confirmation with pharmacy staff throughout all in-person institution visits. To guarantee uniformity across regions, the survey adhered to defined WHO/HAI methods. To record the physical availability of each target medication on a given day, visits were conducted to certain public and private healthcare institutions. A product was designated as “available” if at least one unit of the designated formulation was in stock at the time of assessment. Only community pharmacies in the private sector provided the medication pricing data, which included patient costs for both OBs and LPGs. Since necessary medications are provided at no cost at government institutions across the examined regions, prices were not gathered in the public sector.

### Data Validation

All collected data were carefully checked by the study team for completeness and consistency. Supervisors reviewed the filled survey forms and cross-checked key entries before data analysis to ensure accuracy. Any discrepancies identified during this process were resolved by referring back to the original forms. A double-check procedure was performed for critical variables, including medicine availability and pricing data, to minimize errors [23].

### Data analysis

Data were compiled in a purpose-built spreadsheet developed for this study to capture all raw availability, price, and affordability variables. After cleaning and verification, the dataset was imported into SPSS version 27 (IBM Corp., Armonk, NY, USA) for statistical analysis. All analyses were conducted under three predefined domains: medicine availability, patient prices (private sector only), and affordability [24].

### Availability analysis

Availability was expressed as the proportion of surveyed facilities in which a given medicine was physically present on the day of data collection. Mean availability values were then computed in SPSS separately for public and private sectors across the six surveyed regions, and results were presented for both originator brands (OBs) and lowest-priced generics (LPGs). Following WHO/HAI reporting conventions, availability levels were categorized as absent (0%), very low (< 30%), low (30–49%), fairly high (50–80%), and high (> 80%) [13]. The categories were employed for descriptive classification only in accordance with WHO/HAI reporting conventions and were not used in inferential statistical modeling.

### Statistical modeling

For the regression analysis, each medicine at each facility was treated as a separate observation and medicine availability was defined at the facility-medicine level. Medicine availability was the outcome variable and was coded as a binary outcome (1 = available if at least one unit of the medicine was found in a given facility on the day of data collection and 0 = not available if there was no stock). The independent variables were sector (public vs. private), brand type (originator brand (OB) vs. lowest-priced generic (LPG)), level of healthcare (primary, secondary, tertiary) and geographic area (district). We fitted a multivariable logistic regression model to estimate odds ratios (ORs) adjusted for 95% confidence intervals (CI). The reference categories were public sector, originator brands, primary health care facilities and Islamabad. The overall significance of categorical variables with more than 2 categories (healthcare level and district) was evaluated using the Wald chi-square test. Statistical significance was defined as a p-value < 0.05.

### Price analysis

To guarantee statistical robustness, median unit prices (MUPs) were computed for every medication offered in at least three private-sector institutions; medications with fewer observations were not included in MUP analysis. The 2015 Management Sciences for Health (MSH) International Drug Price Indicator Guide provided the International Reference Prices (IRPs). To maintain temporal comparability with locally collected price data, the MSH 2015 IRPs were inflated to the study year using the Drugs and Medicines Consumer Price Index (CPI) published by the Pakistan Bureau of Statistics, rather than deflating local prices. Medicines lacking a corresponding IRP in the MSH guide were excluded from MPR calculations but were retained for availability and local price analyses [24]. This comparison yielded the Median Price Ratio (MPR), calculated as:

$$\:\mathrm{MPR}=\frac{\mathrm{Median\:Local\:Unit\:Price}}{\mathrm{International\:Reference\:Unit\:Price}}$$

Values larger than 1.0 imply that the local price is higher than the international benchmark, while an MPR of 1.0 denotes parity with the international reference price [24].

### Affordability analysis

The cost of a typical treatment course was compared to the daily salary of Pakistan’s lowest-paid unskilled government worker (LPGW) to determine affordability. For 2025, the daily wage was set at PKR 1333.33 (approximately US$ 4.78). The number of days’ income needed to pay for a 30-day maintenance therapy for diabetes or chronic cardiovascular diseases was used to measure affordability. Affordable treatments were those that cost no more than one day’s salary. The World Health Organization Defined Daily Dose (WHO DDD) served as the basis for the normal daily doses utilized in affordability calculations; doses suggested by current clinical treatment guidelines were added as needed.

$$\eqalign{ & \>{\rm{Affordability}}\>{\rm{ = }}\, \cr & {{{\rm{Cost}}\>{\rm{of}}\>{\rm{Standard}}\>{\rm{treatment}}\>{\rm{for}}\>{\rm{1}}\>{\rm{month}}} \over {{\rm{Daily}}\>{\rm{wage}}\>{\rm{of}}\>{\rm{lowest}}\>{\rm{paid}}\>{\rm{unskilled}}\>{\rm{government}}\>{\rm{worker}}}} \cr} $$

If the result is ≤ 1 day, the treatment is considered affordable. If it is > 1 day, it is considered unaffordable.

## Results

### Medicine availability

**Fig. 1:**
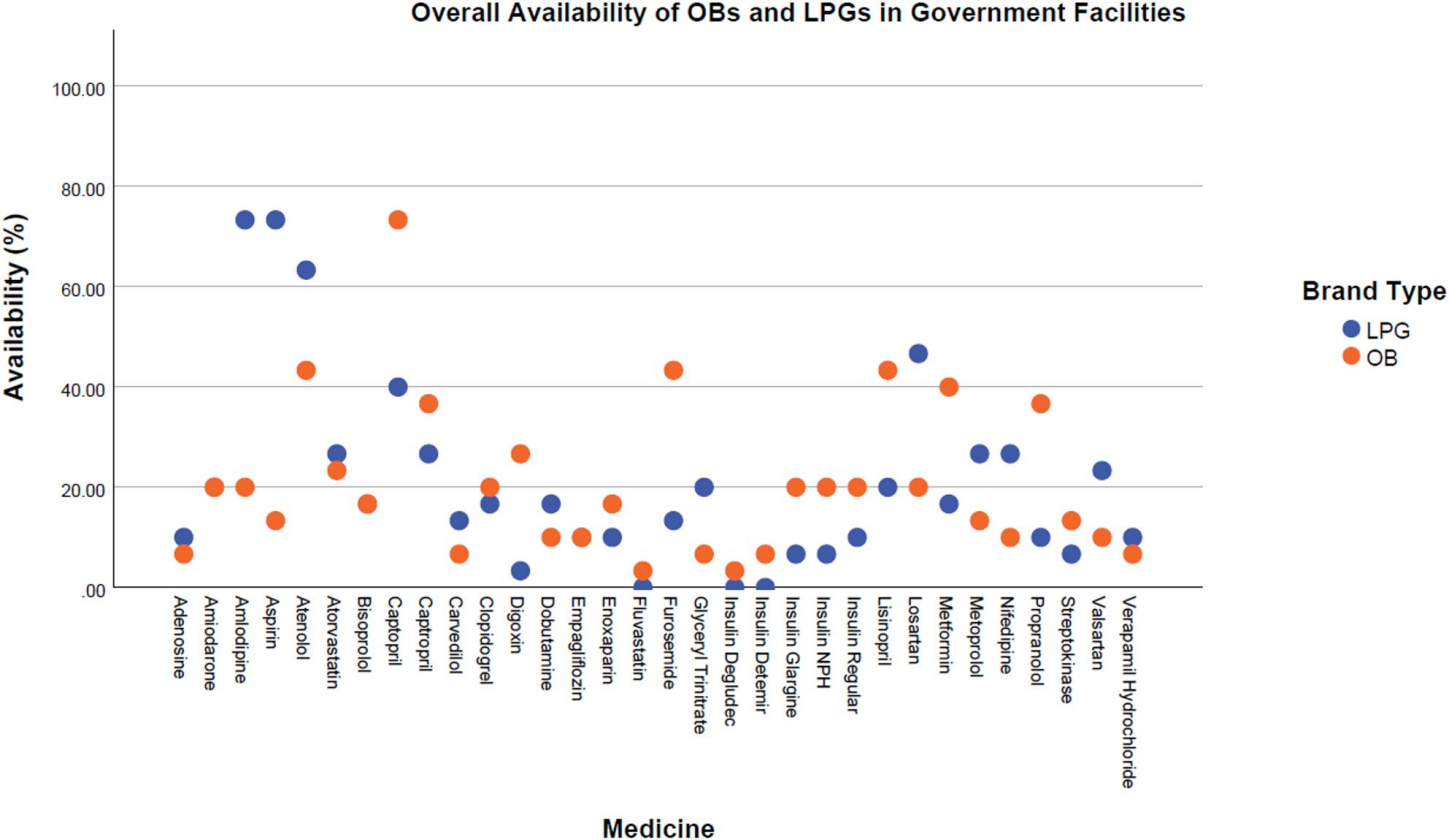
Availability of originator brands (OBs) and lowest-priced generics (LPGs) for selected medicines in government sector facilities

**Fig. 2:**
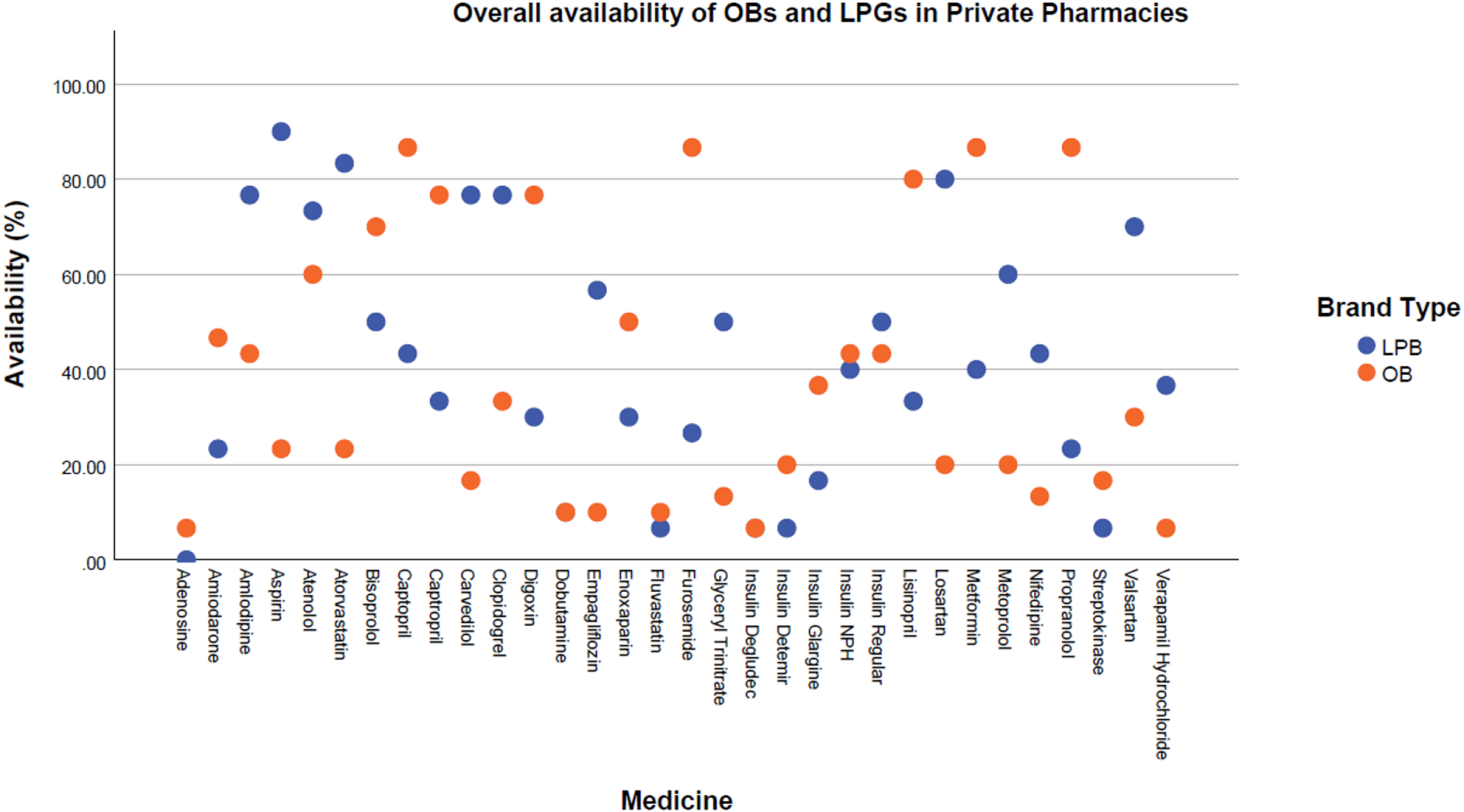
Availability of originator brands (OBs) and lowest-priced generics (LPGs) for selected medicines in private sector pharmacies

The availability of the 32 cardiovascular and diabetes medications was generally low, with significantly worse access in government facilities, where many medications were only 50% available and some, like several generic insulin analogs, were completely unavailable. Several first-line treatments were more readily available in private pharmacies, with some LPGs reaching 80–92% and several OBs around 84%. But essential emergency medications (e.g. the streptokinase and adenosine) were still in short supply in both industries. The availability of insulin was inadequate in all sectors and only slightly improved in private pharmacies (Figs. 1 and 2).

**Fig. 3:**
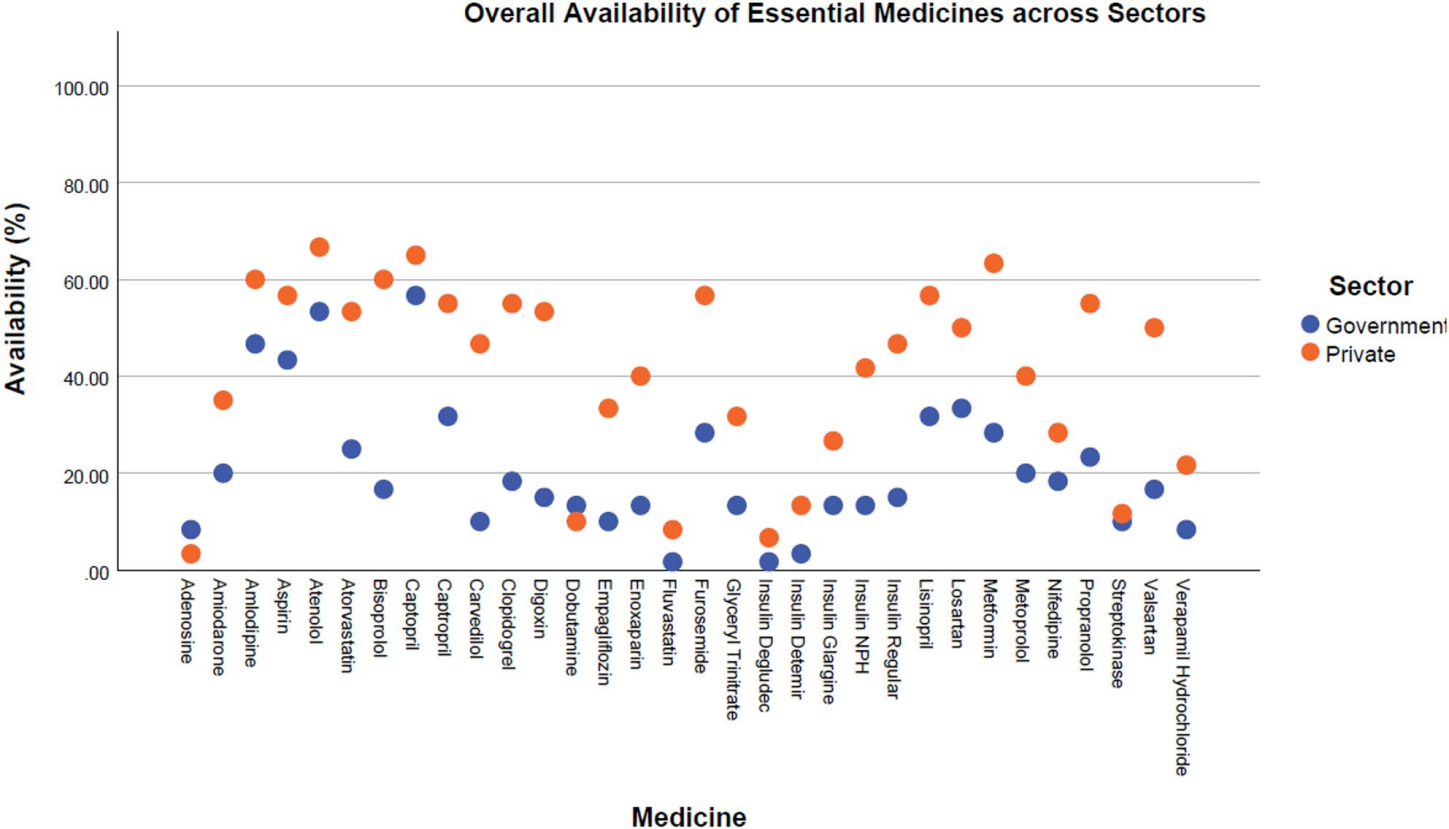
Availability of selected medicines across government and private sector facilities

Figure 3 illustrates the proportion of vital cardiovascular and diabetic drugs that are offered across the public and private sectors. For many medications, private pharmacies are consistently more accessible than government facilities, demonstrating a glaring sectoral disparity in access. Several first-line treatments (e.g. aspirin, amlodipine, atorvastatin, and certain antihypertensives) exhibit moderate-to-high availability in the private sector, while the corresponding availability in government facilities is frequently significantly lower. Even though private stores are performing better, many medications, especially emergency and specialty items, remain below the WHO’s ideal target of 80% availability [25] (e.g. adenosine, streptokinase, and certain insulin preparations), indicating ongoing deficiencies in supply. A chi-square test of independence revealed a strong correlation ($\:{\chi\:}^{2}\left(1\right)=180.568$, *p* < 0.001) between the availability of medicines and the healthcare sector (*n* = 3840).

**Fig. 4:**
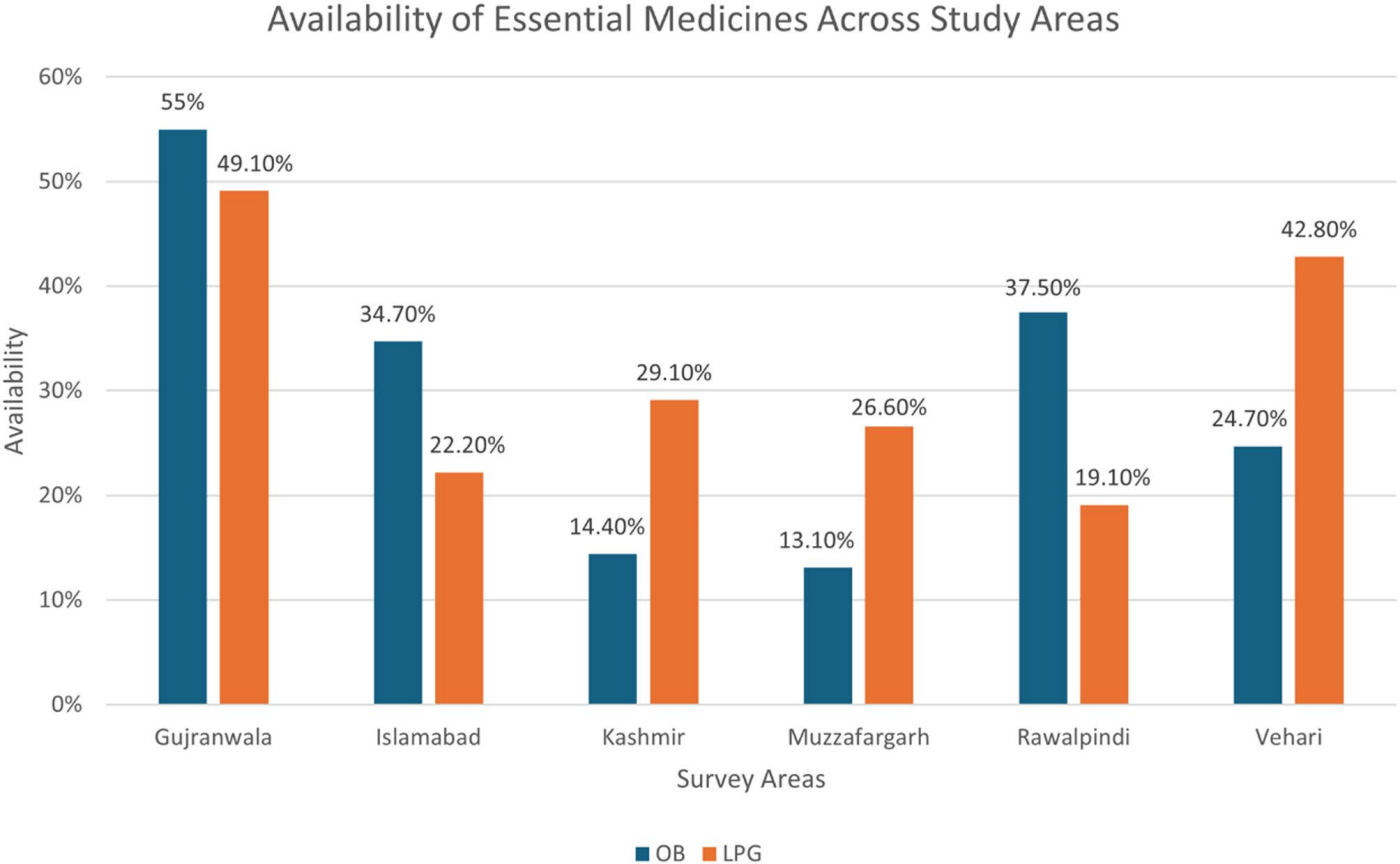
Comparative availability of Originator Brand (OB) and Lowest- Price Generic (LPG) medicines across six surveyed areas in Pakistan

There was considerable variance in medicine availability among the examined areas, indicating major regional disparities in access to important medicines (Fig. 4). With Originator Brands (OB) accessible at 55.0% and Lowest Price Generics (LPG) at 49.1%, Gujranwala outperformed other areas and showed the highest overall availability. On the other hand, Originator Brands were alarmingly scarce in more remote or resource-constrained areas like Muzaffargarh and Kashmir (13.1% and 14.4%, respectively), whereas Lowest Price Generics were somewhat more accessible in these locations (26.6% and 29.1%). It’s interesting to note that major cities like Rawalpindi and Islamabad displayed moderate availability levels, with a noticeable preference for Originator Brands (34.7% and 37.5%) over Generics (22.2% and 19.1%), potentially indicating market dynamics driven by brand preference in affluent urban settings. On the other hand, Vehari showed a clear trend in which generic medications (42.8%) were significantly more accessible than original brands (24.7%), indicating a market dependence on less expensive substitutes in this district (Fig. 4). A chi-square test of independence demonstrated a significant correlation between district and medication availability ($\:{\chi\:}^{2}\left(5\right)=202.781$, *p* < 0.001, *n* = 3840), with no predicted cell counts below 5, hence meeting the requirements for the test.

### Affordability

OBs and LPGs were clearly distinguished by the affordability analysis, which was based solely on patient costs gathered from private-sector retail pharmacies. When purchased as LPGs, most essential cardiovascular and antidiabetic drugs were fairly priced; a typical 30-day course of therapy often cost less than one day’s wages of the lowest-paid unskilled government employee. Conversely, OBs often imposed higher charges, with some drugs approaching or surpassing the one-day wage threshold, indicating a greater financial burden for patients who rely on branded prescriptions. LPGs continued to be far less expensive than OBs for frequently used oral medications, frequently much less than one day’s pay. Many first-line treatments remained somewhat accessible despite the somewhat higher cost of OBs; nevertheless, for other medications, OB treatment prices exceeded the affordability barrier while LPGs stayed within reasonable bounds. This pattern emphasizes the need of encouraging LPG usage in standard clinical practice and the significant potential for cost reductions through generic substitution. An important exception to this general pattern was insulin treatments. When acquired as OBs, insulin analogues, especially pen formulations, were linked to a significant cost burden, needing several days’ income for a 30-day course of treatment. When compared to oral antidiabetic medications, even LPG versions of insulin analogs continued to be somewhat costly. Human insulin formulations provided in vials, on the other hand, were significantly less expensive, typically costing less than one day’s salary. Overall, these results show that although most necessary medications are reasonably priced in the private sector in their generic forms, the persistent reliance on OBs, particularly for insulin analogues, poses a serious financial issue and may limit access for patients with low incomes. The affordability of a selected medications is shown in Table 2 as the number of daily salaries required for a 30-day course of therapy.

**Table 2:** Affordability of selected essential cardiovascular and antidiabetic medicines in Pakistan, expressed as the number of days’ wages required by the lowest-paid unskilled government worker to purchase a 30-day course of treatment

Medicine	Treatment Period (Days)	Units per 30 Days	Median price (OB, PKR)	Median price (OB, USD)	Median price (LPG, PKR)	Median price (LPG, USD)	OB Affordability (Days’ Wages)		LPG Affordability (Days’ Wages)
Aspirin	30	30	150	0.53	78	0.27		0.11	0.06
Atenolol	30	30	480	1.68	240	0.84		0.36	0.18
Atorvastatin	30	30	1200	4.2	930	3.25		0.9	0.7
Captopril	30	60	1404	4.91	900	3.15		1.05	0.68
Captopril	30	30	1217.7	4.26	693	2.42		0.91	0.52
Propranolol	30	120	698.4	2.44	600	2.10		0.52	0.45
Nifedipine	30	30	1050	3.67	384	1.34		0.79	0.29
Lisinopril	30	30	1024.2	3.58	362.1	1.27		0.77	0.27
Losartan	30	30	1350	4.73	720	2.52		1.01	0.54
Metoprolol	30	30	795	2.78	324.9	1.13		0.6	0.24
Amlodipine	30	30	501	1/75	330	1.15		0.38	0.25
Valsartan	30	30	1950	6.83	586.2	1.98		1.46	0.44
Verapamil hydrochloride	30	180	3240	11.34	648	2.27		2.43	0.49
Amiodarone	30	30	984	3.44	918	3.21		0.74	0.69
Bisoprolol	30	60	1494.6	5.23	940.8	3.29		1.12	0.71
Carvedilol	30	60	2520	8.82	2340	8.19		1.89	1.76
Furosemide	30	30	126.6	0.44	120	0.42		0.09	0.09
Digoxin	30	30	108	0.38	109.8	0.38		0.08	0.08
Clopidogrel	30	30	1356.3	4.74	702	2.45		1.02	0.53
Fluvastatin	30	90	4500	14.40	2520	8.82		3.38	1.89
Metformin	30	120	490.8	1.72	480	1.68		0.37	0.36
Empagliflozin	30	30	1800	6.30	1050	3.80		1.35	0.79
Insulin Degludec	30	3 pens	13,500	47.25	2205	7.72		10.13	1.65
Insulin Detemir	30	3 pens	8214	28.74	5028	17.60		6.16	3.77
Insulin Glargine	30	3 pens	4839	16.94	3690	12.92		3.63	2.77
Insulin NPH	30	1 vial	1194	4.17	950	3.32		0.9	0.71
Insulin Regular	30	1 vial	1150	4.02	900	3.15		0.86	0.68

**Notes**: OB = Originator Brand; LPG = Lowest-Priced Generic; PKR = Pakistani Rupee; USD = United States Dollar; Affordability calculated as number of days’ wages of the lowest-paid government worker required to purchase a 30-day treatment; Prices in USD converted using exchange rate of 1 PKR = 0.0035 USD (as of October 1, 2025)

### Patient prices in the private sector

Analysis of patient prices collected exclusively from private-sector retail pharmacies revealed a consistent and marked price differential between OBs and LPGs. Patients who were unable to get LPGs had a significant financial hardship since OBs were consistently more expensive than their generic counterparts across all studied medications. Although LPGs offered clear cost advantages, their prices remained variable across therapeutic classes, with medicines used for chronic conditions, particularly diabetes and cardiovascular diseases, showing higher median treatment costs compared with commonly used acute therapies (Table 3).

**Fig. 5:**
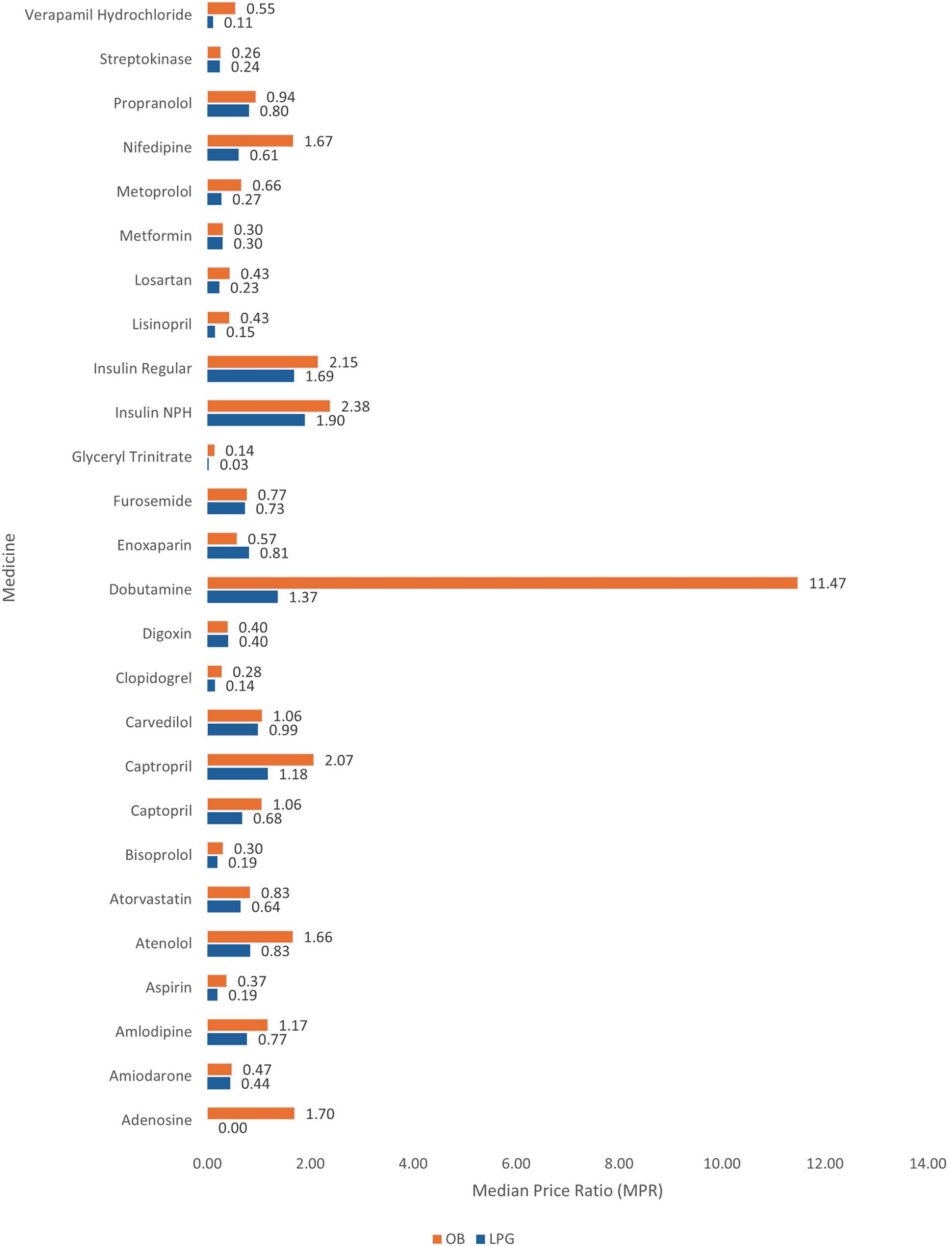
Comparison of median price ratios for originator brands and lowest-priced generics in private pharmacies

Price disparities were most pronounced for newer and branded medicines, where OB prices exceeded international reference prices, while LPGs, although lower, remained moderately priced. In contrast, older, off-patent medicines demonstrated relatively narrower price gaps between OBs and LPGs, reflecting greater market competition. Importantly, several medicines that appeared affordable at the unit level accumulated considerable monthly costs due to long-term use, reinforcing the relevance of assessing prices in the context of standard treatment regimens rather than per-unit costs alone. Given the limited availability of medicines in the public sector, private-sector patient prices represent the primary determinant of out-of-pocket expenditure for a large proportion of patients. The observed price patterns indicate that, despite existing price regulation mechanisms, private-sector pricing continues to pose a barrier to equitable access, particularly for patients requiring long-term or lifelong therapy. Median price ratios (MPRs) for both OBs and LPGs further illustrate these pricing differentials and are presented in Fig. 5, highlighting the persistent cost disadvantage associated with originator brands compared with generics.

**Table 3:** Average prices of essential cardiovascular and antidiabetic medicines by therapeutic class in public and private sectors (PKR and USD)

Main Therapeutic Category	Sub-Class	Medicines Included	OB Price (PKR)	OB price (USD)	LPG Price (PKR)	LPG Price (USD)
Antihypertensives	ACE Inhibitors	Captopril, Lisinopril	1201.95	4.21	630.50	2.21
	Angiotensin Receptor Blockers (ARBs)	Losartan, Valsartan				
	β-blockers	Atenolol, Propranolol, Metoprolol, Bisoprolol, Carvedilol				
	Calcium Channel Blockers (CCBs)	Nifedipine, Amlodipine, Verapamil HCl				
	Diuretics	Furosemide				
Antidiabetics	Biguanides	Metformin	4455.40	15.60	2043.29	7.15
	SGLT-2 Inhibitors	Empagliflozin				
	Insulins (All types)	Degludec, Detemir, Glargine (Long), NPH (Intermediate), Regular (Short)				
Dyslipidemic Agents	Statins	Atorvastatin, Fluvastatin	2850	9.97	1725	6.03
Antithrombotics	Antiplatelet Agents	Aspirin, Clopidogrel	753.15	2.63	390	1.36
Other Cardiac Agents	Antiarrhythmics	Amiodarone	984	3.44	918	3.21
	Cardiac Glycosides	Digoxin	108	0.38	109.8	0.38

Notes: OB = Originator Brand; LPG = Lowest-Priced Generic; Prices represent median cost of medicines in each subclass; USD prices calculated at 1 PKR = 0.0035 USD (October 1, 2025); Medicines grouped by main therapeutic category and subclass for policy relevance

### Factors associated with medicine availability

A multivariate logistic regression was used to find variables related to the availability of medications. Sector (public vs. private), brand type (generic vs. original), healthcare level (primary, secondary, tertiary), and geographic region (district) were all included as factors in the model. To gain a better understanding of systemic inequities across healthcare settings and geographies, this analysis sought to quantify how these characteristics are associated with the likelihood that drugs are accessible. Table 4 summarizes the model’s results, emphasizing important associated factors and their corresponding impacts.

Several important determinants of medication availability were found using multivariable logistic regression analysis. The availability of medicines in the private sector was nearly three times higher than in the public sector (OR = 2.90, 95% CI: 2.49–3.37; *p* < 0.001), indicating significant sectoral differences. Because the availability of the cheapest generics did not differ statistically significantly from that of the original brands (OR = 1.09, 95% CI: 0.94–1.26; *p* = 0.260), brand type was not a significant factor associated with availability. Availability was strongly impacted by healthcare level. Primary healthcare facilities were used as the reference category (OR = 1) in the regression model. Secondary (OR = 0.43, 95% CI: 0.36–0.50; *p* < 0.001) and tertiary (OR = 0.44, 95% CI: 0.36–0.54; *p* < 0.001) healthcare institutions had substantially lower odds of medicine availability compared with primary care, indicating decreased access at higher levels of care. There was also clear geographic variance. Gujranwala exhibited significantly greater availability (OR = 3.03, 95% CI: 2.38–3.87; *p* < 0.001) when Islamabad was used as the reference, whereas Vehari showed a slight but significant increase (OR = 1.31, 95% CI: 1.02–1.68; *p* = 0.032). On the other hand, the odds of availability were significantly lower in Kashmir (OR = 0.68, *p* = 0.004) and Muzaffargarh (OR = 0.60, *p* < 0.001). Islamabad and Rawalpindi did not differ significantly (*p* = 0.948). Overall, the results show clear disparities in medicine availability at the sectoral, healthcare, and district levels.

**Table 4:** Logistic regression showing associations of sector, brand type, healthcare level, and district with availability

Variable	B	SE	Wald	df	*p*-value	Exp(B)	95% CI for Exp(B)
Sector							
Public	1						
Private	1.064	0.077	189.828	1	< 0.001	2.897	2.490–3.370
**Brand Type**							
OB	1						
LPG	0.085	0.075	1.27	1	0.26	1.088	0.939–1.261
**Healthcare Level**			124.467	2	< 0.001		
Primary	1						
Secondary	-0.856	0.085	102.079	1	< 0.001	0.425	0.360–0.502
Tertiary	-0.822	0.106	59.662	1	< 0.001	0.44	0.357–0.541
**District**			206.512	5	< 0.001		
Islamabad	1						
Gujranwala	1.11	0.124	79.537	1	< 0.001	3.033	2.377–3.870
Kashmir	-0.39	0.135	8.3	1	0.004	0.677	0.520–0.883
Muzaffargarh	-0.512	0.138	13.851	1	< 0.001	0.599	0.458–0.785
Rawalpindi	-0.008	0.13	0.004	1	0.948	0.992	0.769–1.278
Vehari	0.272	0.127	4.612	1	0.032	1.313	1.024–1.683
**Constant**	-1.117	0.114	95.208	1	< 0.001	0.327	–

B = coefficient, SE = standard error, Wald = test statistic, Exp(B) = odds ratio, 95% CI = confidence interval. Reference categories: Primary healthcare level and Islamabad district

## Discussion

This study demonstrates persistent and interrelated challenges in the availability and affordability of essential cardiovascular and antidiabetic medicines across Rawalpindi, Islamabad, Gujranwala, Muzaffargarh, Vehari, and Azad Jammu & Kashmir, highlighting a critical gap between national policy commitments and actual access at the point of care. No medicines reached the WHO-recommended availability benchmark of 80% in the government sector for both OBs and LPGs, and several medicines reached or exceeded 80% availability in the private sector for either OBs or LPGs (but not for both brand types for the same medicine), a pattern consistent with past WHO/HAI surveys in Pakistan and other LMICs [24, 26, 27]. Given the rising burden of cardiovascular disease and diabetes in Pakistan, inadequate access to these medicines poses a serious public health threat [28]. A concerning inverse relationship between availability and affordability was observed, wherein LPGs were generally more affordable but less available, while OBs were relatively more available yet imposed a higher financial burden. This pattern suggests market-driven stocking and prescribing practices that favor branded medicines, often influenced by pharmaceutical promotion and profit margins, rather than patient affordability [29, 30]. Although several oral cardiovascular medicines appeared affordable when assessed individually using the standard WHO/HAI benchmark of one day’s wage, this likely underestimates the true economic burden, as patients with chronic diseases frequently require multiple medicines over long periods [31, 32]. The affordability crisis was most pronounced for insulin therapies, particularly insulin analogues, which required multiple days’ wages for a standard monthly treatment while simultaneously exhibiting exceptionally low availability. This dual barrier is especially alarming in the context of Pakistan’s rapidly increasing diabetes prevalence and the life-sustaining nature of insulin therapy [33]. Even human insulins, which were relatively more affordable, showed inconsistent availability, reflecting systemic weaknesses in procurement, cold-chain logistics, and supply prioritization, issues that have also been documented in other LMIC settings [34].

Marked inter-district disparities further illustrate structural inequities in Pakistan’s health system. Urban centers such as Islamabad and Rawalpindi demonstrated comparatively better availability, particularly of OBs, likely due to stronger purchasing power, higher patient turnover, and proximity to pharmaceutical distribution hubs. However, even in these cities, LPG availability remained inconsistent, limiting the potential benefits of generic substitution. In contrast, predominantly rural districts such as Muzaffargarh and Vehari exhibited poorer availability across multiple medicine categories, compounded by higher poverty levels, weaker health infrastructure, and greater dependence on out-of-pocket (OOP) payments [35]. Azad Jammu & Kashmir faced additional logistical constraints related to geography and transport, which likely contributed to poor availability, particularly of temperature-sensitive medicines such as insulin. Gujranwala presented a mixed pattern, suggesting that economic activity alone does not ensure equitable access in the absence of strong regulatory oversight and supply chain governance.

From a regulatory perspective, the findings indicate that existing price control mechanisms enforced by the Drug Regulatory Authority of Pakistan (DRAP) have had limited impact on improving affordability, as they primarily focus on setting maximum retail prices rather than ensuring sustained availability or promoting generics [12]. While price regulation may reduce excessive price variation, it does not address prescriber behaviour, pharmacy stocking incentives, or public perceptions regarding generic medicines. Evidence from Pakistan suggests that misconceptions about the bioequivalence and quality of generics among healthcare professionals and patients continue to undermine generic prescribing and dispensing [36, 37]. The public health implications of these findings are substantial. Pakistan relies heavily on OOP expenditure, accounting for nearly 70% of total health spending, leaving households vulnerable to catastrophic health expenditures (World Health Organization, 2022). Persistent unaffordability and poor availability of essential medicines are likely to result in non-adherence, delayed treatment initiation, and disease progression, ultimately increasing avoidable hospitalizations and premature mortality, particularly in rural and underserved populations [28].

This study has several limitations that should be considered. First, data collection was conducted at a single point in time, which does not capture seasonal fluctuations or temporal changes in stock levels. This is particularly important in districts such as Muzaffargarh and Azad Jammu & Kashmir, where supply chains are often unstable and stock-outs of essential medicines, including temperature-sensitive products like insulin, are common. Second, although clear inter-district disparities were observed, this study did not examine the underlying factors contributing to these differences, such as variations in provincial procurement strategies, logistics management, or local governance. In terms of affordability, using the wage of the lowest-paid government worker as a benchmark likely underestimates the actual financial burden on informal workers and unemployed populations. Moreover, this approach does not account for the cumulative costs of multidrug therapy, which is typical for patients managing cardiovascular disease and diabetes, meaning out-of-pocket expenses are likely higher than indicated by single-medicine estimates. Finally, while the selected districts represent a range of geographic and socioeconomic contexts, the underrepresentation of remote rural areas may limit the generalizability of these findings to the broader Pakistani population. Additionally, a lack of maintained or comprehensive databases providing regional or country-specific statistics on access to essential medicines meant that some claims had to rely on global-level references, representing another limitation of this study.

## Recommendations

Several policy-relevant actions emerge from this evidence. Strengthening NEML-based procurement with enforceable minimum stock requirements is essential, particularly for medicines used in chronic disease management. Mandatory generic prescribing and pharmacist-led substitution policies, supported by targeted training and public education campaigns, could substantially reduce OOP expenditures and improve affordability [32]. Innovative financing mechanisms, including insurance schemes that explicitly cover outpatient medicines, are urgently needed to protect middle- and low-income households. In addition, routine monitoring of medicine availability and prices using standardized digital tools, such as the WHO Essential Medicines and Health Products Price and Availability Monitoring (MedMon) system, could improve transparency and accountability. Promoting local production of essential medicines through fiscal incentives and tax exemptions may further enhance supply resilience and reduce dependency on imports.

## Conclusion

This study reveals significant gaps in access to essential cardiovascular and antidiabetic medicines in six districts of Pakistan, showing a disconnect between policy and actual availability. No government sector medicines met the WHO’s 80% availability benchmark, while some private sector medicines did, mainly brand-name ones. The relationship between availability and affordability is problematic: generics are cheaper but less available, whereas branded medicines are more accessible but costlier. This is particularly concerning for insulin therapies, which are both low in availability and high in cost, posing challenges for managing chronic diseases amid a rising prevalence of diabetes.

Marked inter-district disparities highlight structural inequities in Pakistan’s health system, with urban centers having better health resources compared to rural areas and regions like Muzaffargarh and Azad Jammu & Kashmir. These areas face ongoing shortages exacerbated by inadequate infrastructure and poverty. Regulatory measures, such as DRAP’s price controls, are ineffective as they fail to influence prescribing practices, pharmacy incentives, or public views on generic medicines.

The public health implications of unaffordable and poorly available essential medicines are profound, leading to treatment non-adherence and disease progression, particularly among underserved populations. Solutions require a multifaceted policy approach that includes enforceable procurement, mandatory generic prescribing, targeted education campaigns, and support for local medicine production. Inadequate access to essential medicines remains a significant barrier to managing non-communicable diseases in Pakistan, necessitating coordinated efforts beyond mere price regulation to ensure equitable access and mitigate escalating health and economic costs associated with conditions like cardiovascular disease and diabetes.

## Data Availability

The datasets generated and analysed during the current study are available from the corresponding author on reasonable request.
